# Genome-wide identification and expression analysis of the glycosyl hydrolase family 1 genes in *Medicago sativa* revealed their potential roles in response to multiple abiotic stresses

**DOI:** 10.1186/s12864-023-09918-w

**Published:** 2024-01-02

**Authors:** Haiming Kong, Jiaxing Song, Shihai Ma, Jing Yang, Zitong Shao, Qian Li, Zhongxing Li, Zhiguo Xie, Peizhi Yang, Yuman Cao

**Affiliations:** 1 College of Grassland Agriculture, Northwest A&F University, Yangling, 712100 Shaanxi China; 2 College of Grassland and Environment Sciences, Xinjiang Agricultural University, Urumqi, 830052 China; 3 Shaanxi Academy of Forestry, Xi’an, 710082 China

**Keywords:** GH1 family, Alfalfa, Genome-wide, Gene structure, Abiotic stress, Expression pattern

## Abstract

Glycoside hydrolase family 1 (GH1) β-glucosidases (BGLUs), are encoded by a large number of genes, which participate in the development and stress response of plants, particularly under biotic and abiotic stresses through the activation of phytohormones. However, there are few studies systematically analyzing stress or hormone-responsive *BGLU* genes in alfalfa. In this study, a total of 179 *BGLU* genes of the glycoside hydrolase family 1 were identified in the genome of alfalfa, and then were classified into five distinct clusters. Sequence alignments revealed several conserved and unique motifs among these MsBGLU proteins. Many *cis*-acting elements related to abiotic stresses and phytohormones were identified in the promoter of some *MsBGLUs*. Moreover, RNA-seq and RT-qPCR analyses showed that these *MsBGLU* genes exhibited distinct expression patterns in response to different abiotic stress and hormonal treatments. In summary, this study suggests that *MsBGLU* genes play crucial roles in response to various abiotic stresses and hormonal responses, and provides candidate genes for stress tolerance breeding in alfalfa.

**Supplementary Information:**

The online version contains supplementary material available at 10.1186/s12864-023-09918-w.

## Introduction

 Plants are exposed to various environmental stresses during their growth and development processes, including biotic and abiotic stresses, such as pathogens, salt stress, drought stress, and cold stress, etc. [1, 2], which seriously affect the yield and quality of crops [3]. To adapt to various stresses, plants produce a variety of defensive chemicals by coordinating a complex network of genes [4]. The glucosylated forms of several defensive chemicals include cyanogenic, isoflavone, and hydroxamic acid glucosides. In addition to protecting plants against harmful effects on their defense system, glucosylation may increase the solubility and stability of these chemicals, making them appropriate for storage in the vacuole or other organelles [5, 6]. Glycoside hydrolases (EC 3.2.1) belong to glycosylases (EC 3.2), which are a major class of hydrolases (EC 3). They are classified into a group of enzymes that hydrolyze the glycosidic bonds of carbohydrates [7]. So far, 161 families have been identified and classified in the CAZy (Carbohydrate-Active enZYmes) database [8, 9]. Among these families, the glycoside hydrolase family 1 is recognized for its β-glycosidase activity, which largely contributes to various developmental processes and stress responses in plants [10, 11]. *BGLUs* are groups of glycoside hydrolase 1 (GH1) family members, which can be found in all domains of living organisms, and have the essential function of removing non-reducing terminal glucosyl residues from glycosyl esters, oligosaccharides, and glycosides [9]. A large number of glycosidases have been found in higher plants, which play a variety of abundant functions, including defense, phytohormone activation, cell wall remodeling, scent release, microbe/insect interactions, and secondary metabolism [11, 12].

Several BGLU enzymes have been elucidated to be involved in multiple biological processes of plant growth and development, such as cell wall remodeling, lignification, and plant secondary metabolites [12, 13]. For example, *AtBGLU20* plays a crucial part in pollen formation, as evidenced by the fact that knockdown mutants of *AtBGLU20* have defective pollen grains but normal morphological growth [14]. In *Cymbidium sinense*, *BGLU* genes are upregulated during ovule development, when β-glucosidase may mediate hydrolysis of cellulose [15]. More importantly, substantial evidence also indicates that *BGLU* genes play important roles in the activation of defense compounds response to stresses and in the release of active plant phytohormones [16]. For instance, two stem-specific β-glucosidases, *AtBGLU45* and *AtBGLU46*, are involved in the hydrolysis of monolignol glucoside coniferin, which is the storage form of monolignols. When lignin needs to be synthesized de novo under various stresses, glucoside coniferin in *Arabidopsis thaliana* could be used [17]. Besides, it has been demonstrated that *AtBGLU26* plays an important role in plant protection against broad spectrum fungi in *A*. *thaliana* [18]. When *A*. *thaliana* was treated with salt and drought stress, *AtBGLU48* protected plants against freezing. *AtBGLU48* could increase cold, salt, and drought resistance in *A. thaliana* by protecting chloroplast membranes and preventing dehydration [19]. During dehydration, *AtBGLU18*, has the function of increasing ABA, and increased molecular weight under drought. Compared with wild-type plants, the mutants of *AtBGLU18* exhibit earlier germination, defects in stomatal closure, and increased sensitivity to drought stress [20]. In *Oryza sativa*, *OsBGLU10*, *OsBGLU24*, and *OsBGLU33* are associated with IAA and ABA signaling and play a particular role in seed germination, root elongation, and drought tolerance [21]. Taken together, all these studies suggest that *BGLUs* play an important role in plant growth, development, and stress resistance.

The *BGLU* gene members of the *GH1* family have been identified in several plants through the genome sequencing, and their functions have been preliminarily analyzed. Alfalfa (*Medicago sativa* L.), well-known as the “Queen of forages”, is a perennial legume forage crop, due to its advantages of rich content in protein, vitamins, and other nutrients [22]. However, the growth and development of *M. sativa* were constrained by multiple environmental stress factors, such as drought, salt, and extreme temperature stresses [23, 24]. Therefore, it is essential to investigate the abiotic stress-responsive mechanism and screen new genes for genetic breeding of *M. sativa*. With the rapid development of sequencing technology, a large number of genomes and publicly available transcriptomic data have been collected. Genome-wide identification and analysis of *BGLU* genes have been conducted in many plants. However, the reference genome of alfalfa (cv. XinJiangDaYe) was not published until 2020 [25]; therefore, the basic information and functions of *BGLU* genes in alfalfa have not yet been clarified.

In this study, a total of 179 *MsBGLU* genes from the *GH1* family were identified and characterized. We compared the protein sequences of *A. thaliana* and *Medicago truncatula* to the reference genome of alfalfa using BLAST method. At the same time, HMM method was used to find glyco_hydro_1 domain in The reference genome of alfalfa. Both of the results were intersected, and CD-search was used to examine, resulting in the identification of 179 *MsBGLU* genes. The comprehensive analyses on these *MtBGLUs* were performed, including sequence features, phylogenetic relationships, gene structures, motif composition, conserved domains, chromosomal localization, synteny analysis, and *cis*-acting elements in the promoter regions, and the evolutionary relationship between *M*. *sativa*, *A*. *thaliana*, *M*. *truncatula*, and *Glycine max*. In addition, *MsBGLUs* were found to participate in various biological processes by analyzing the expression patterns under cold, salt, drought, and ABA treatment, with the public transcriptomic data, as well as checked by quantitative real-time PCR (qRT-PCR) analysis. This study establishes the foundation for a deeper understanding of the functional characteristics of members in the *MsBGLUs* family and their utilization in molecular breeding to enhance resistance against various abiotic stresses in alfalfa.

## Results

### Identification of *BGLU* genes in the *M. sativa* genome

A total of 179 *MsBGLU* genes of the *GH1* family were obtained by HMM and BLAST in the alfalfa (cv. XinJiangDaYe) genome after the removal of redundant sequences. *MsBGLU1* to *MsBGLU179* were named based on their locations on the alfalfa chromosomes. Physico-chemical properties of *MsBGLU* genes were listed in Supplementary Table S1, including instability index, aliphatic index, molecular weights, isoelectric points, average of hydropathicity (GRAVY), and possible subcellular localization. The lengths of the predicted precursor proteins ranged from 276 (*MsBGLU23*) to 1521 (*MsBGLU16*) amino acids (AAs), corresponding to protein molecular weights (MWs) ranging from 31.4 to 171.2 kDa, and the predicted pI ranged from 5.05 (*MsBGLU125*) to 9.34 (*MsBGLU23*). The GRAVY values were all negative except *MsBGLU19*, indicating that most of the MsBGLU proteins were hydrophilic. Based on the subcellular localization predictions, 56 (75.61%) proteins were found in the cytoplasm, 46 (25.70%) in the chloroplast, and 26 (14.53%) in the vacuole. The remaining proteins were located in the extracellular, endoplasmic reticulum, mitochondrion and nucleus. The results suggest that the MsBGLU proteins may have functions in diverse microcellular environments.

### Multiple sequence alignment, phylogenetic analysis, and classification of *MsBGLU *genes

**Fig. 1:**
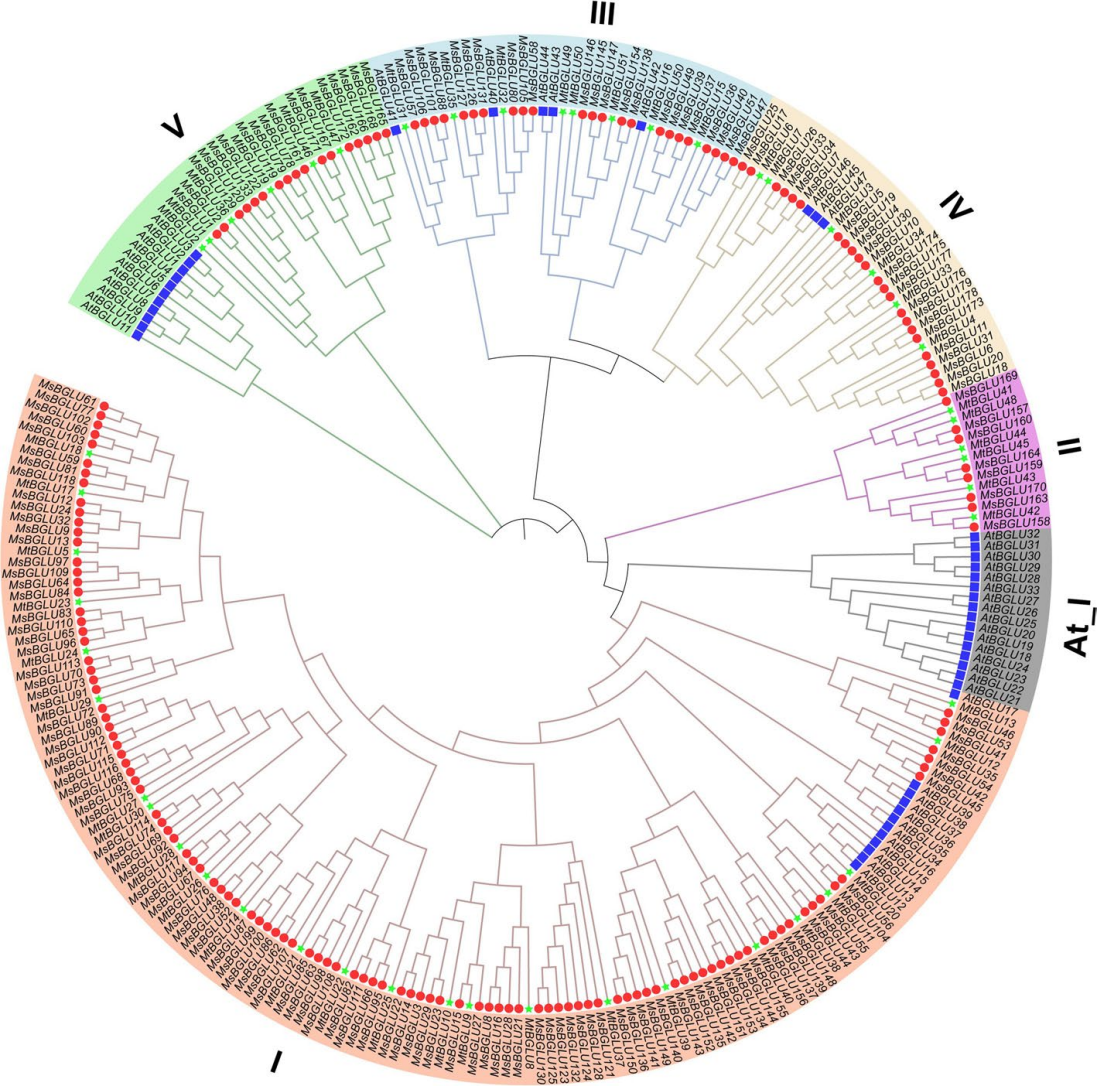
Phylogenetic analysis of *BGLUs* from *M. sativa*, *M. truncatula*, and *A. thaliana*. Full-length protein sequences of BGLUs were used to construct the unrooted tree, by using MEGA-X based on the Neighbor-Joining (NJ) method with a bootstrap value of 1000 replicates. Subfamilies are highlighted with different colors. The red circles, green stars, and blue squares shapes represent BGLU proteins from *M. sativa* (Ms), *M. truncatula* (Mt), and *A. thaliana* (At), respectively

To assess the evolutionary relationships among *MsBGLU* genes, BGLU proteins from *A*. *thaliana*, *M*. *truncatula*, and *M*. *sativa* were used to construct a phylogenetic tree by MEGA-X using the NJ method. Based on the homology and classification of *BGLU* genes in *A*. *thaliana* and *M*. *truncatula*, the 179 MsBGLU proteins were divided into 5 major phylogenetic clusters: I, II, III, IV, and V groups with 108, 8, 23, 23, and 17 members, respectively (Fig. 1). Cluster I was the largest *MsBGLU* subfamily in this study. Cluster II contained 6 *MtBGLUs* and 8 *MsBGLUs*. The At_I Cluster with 16 members was all from *A*. *thaliana*, which was consistent with the previous study on *M*. *truncatula* [26].

### Conserved motif and gene structure analyses of *MsBGLU*

**Fig. 2:**
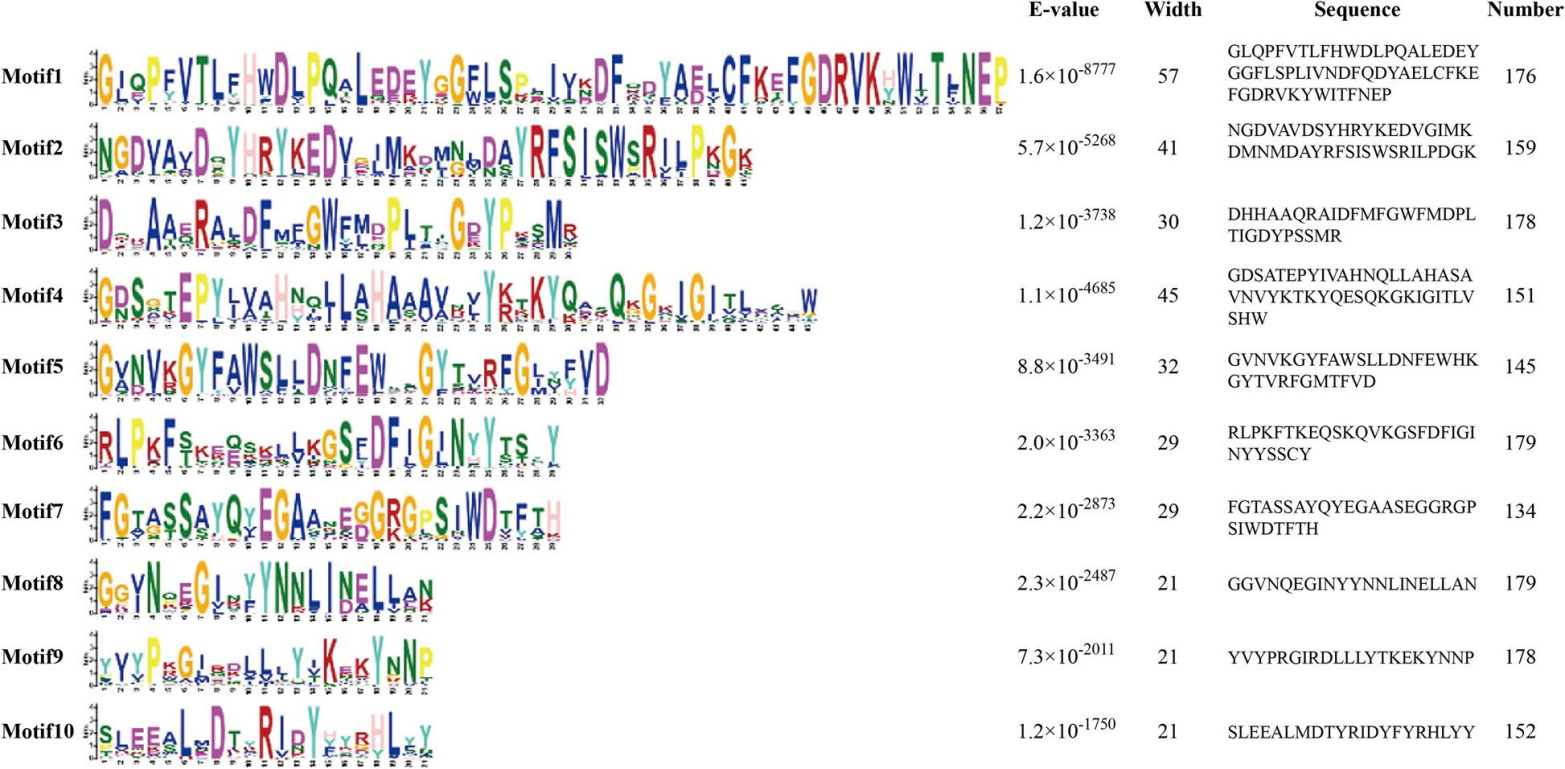
Conserved motif information for MsBGLU proteins

To evaluate the structural similarity and diversification of MsBGLU proteins, a total of 10 different conserved motifs were identified using the MEME tool. The sequences and SeqLogo values of the 10 conserved motifs were presented in Fig. 2. The results showed that the sequence length of the 10 motifs ranged from a minimum of 21 amino acids (Motif8, 9, 10) to a maximum of 57 amino acids (Motif1). Most MsBGLU contained all 10 motifs, indicating their conservation across the gene family. Each member of the MsBGLU gene family contained a minimum of 4 to a maximum of 10 of these motifs (Supplementary Figure S1, Supplementary Table S3); 63%, 23% and 10% of the MsBGLU members contained ten, nine and eight different motifs, respectively. MsBGLU23 contained only four motifs (Motif4, 6, 7, 9); two of MsBGLUs contained only six motifs and four of MsBGLUs contained seven motifs. Conserved motifs of BGLU proteins were located in the glyco_hydro_1 domain, and the distribution of these variable motifs may lead to a diversity of MsBGLU functions.

To understand the gene-structural variations of the *BGLU* family in alfalfa, the compositions of exon-introns were further analyzed using TBtools. The results showed that the number of exons in *MsBGLU* genes ranged from 7 (*MsBGLU115*) to 43 (*MsBGLU16*) (Supplementary Figure S1). Among the 179 *MsBGLUs*, 36%, 16%, 12% of *MsBGLUs* possessed 13, 12, and 14 exons, respectively. These results indicate that the intron/exon distribution of *MsBGLUs* is highly variable, with the structure of 13 exons being the main form of *MsBGLUs* (Supplementary Figure S1).

### Chromosomal distribution and synteny analysis of *MsBGLU* genes

**Fig. 3:**
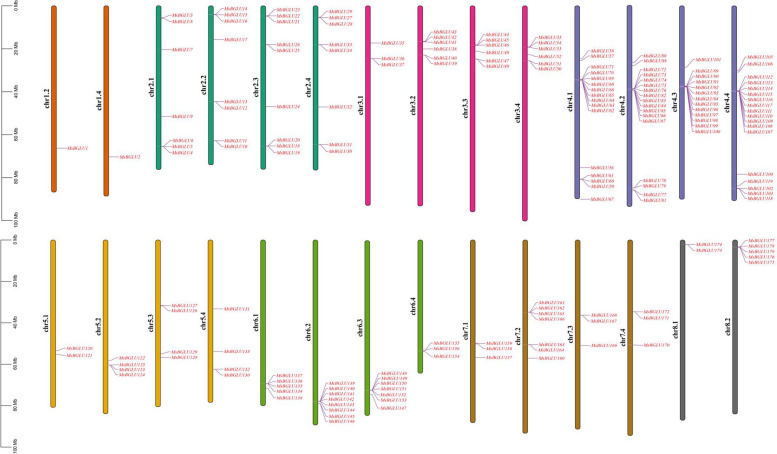
Distribution and position of *MsBGLU* genes across the 28 chromosomes in alfalfa genome. The scale (Mb) represents the lengths of the chromosomes

The 179 members of the *MsBGLU* genes were unevenly distributed across 28 chromosomes of alfalfa reference genome (cv. XinJiangDaYe) [25], except for chr1.1, chr1.3, chr8.3 and chr8.4 (Fig. 3). The chromosome with the highest number of *BGLU* genes (18) was chr4.4, whereas the fewest, only 1, were found on chr1.2 and chr1.4. The *BGLU* gene distributions also presented clustering on some chromosomes, such as *MsBGLU139*-*MsBGLU146* on chr6.2 and *MsBGLU89*-*MsBGLU100* on chr4.3 (Fig. 3).

**Fig. 4:**
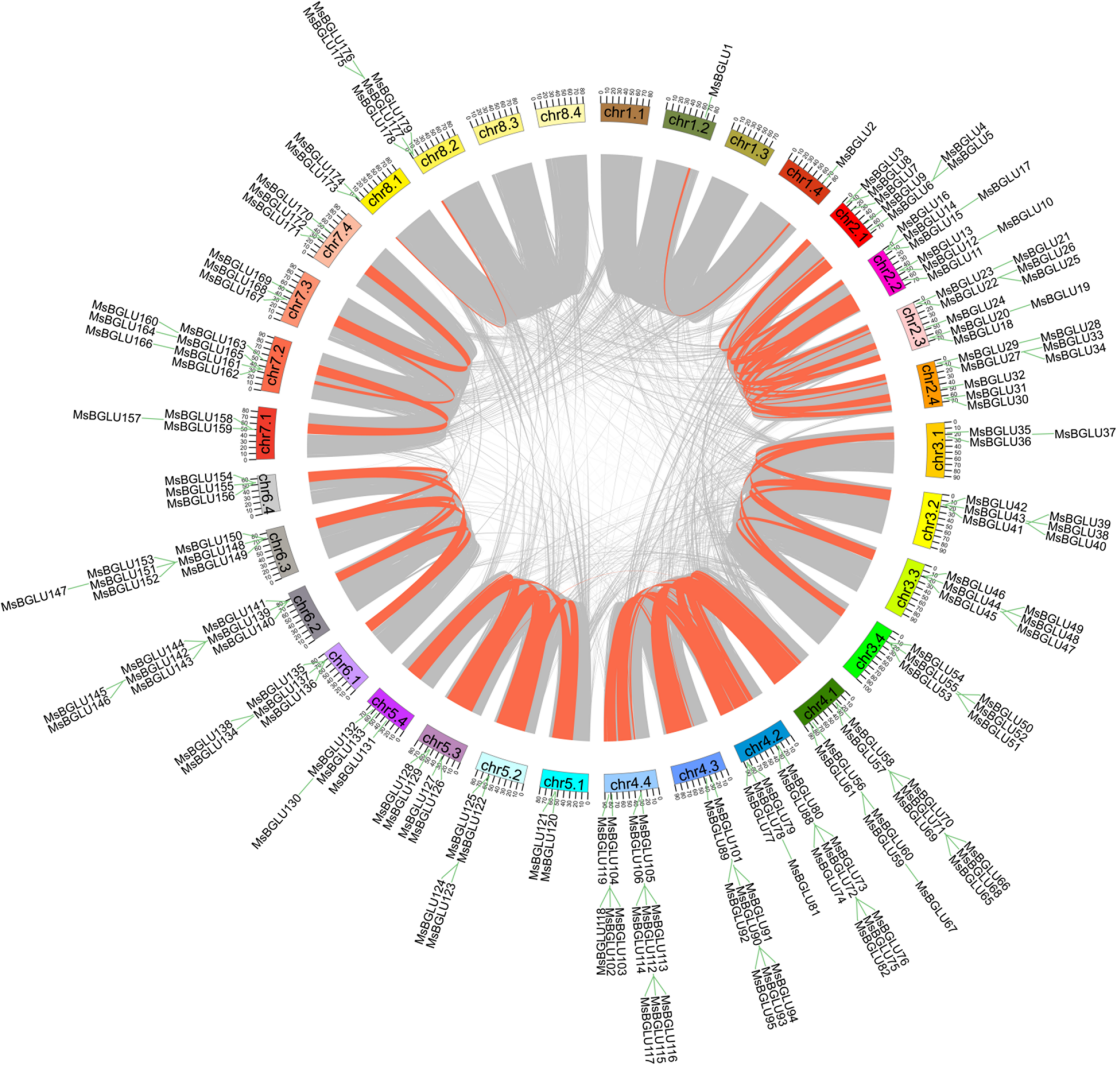
Schematic representation of the interchromosomal relationship among the *MsBGLUs*. Syntenic blocks in the alfalfa genome are indicated by lines in orange

As shown in Fig. 4 and Supplementary Table S3, a total of 150 pairs of homologous genes were found, involving 90 *MsBGLU* members in alfalfa genome. Among these duplication events, 15 were involved in duplications of the four allelic chromosomes. For example, the paralogy of *MsBGLU5* on chr2.1 and *MsBGLU10* on chr2.2, as well as *MsBGLU19* on chr2.3 and *MsBGLU30* on chr2.4, arose from genome duplication events. Among the 179 *MsBGLU* members, 80% *MsBGLUs* belonged to segmental duplications, while 7%, 9%, and 2% of *MsBGLUs* belonged to tandem, proximal and dispersed duplications, respectively (Supplementary Table S3). In addition, no singletons were observed. These results indicate that the segmental duplication is the key driving force in the evolution of *MsBGLUs*.

**Fig. 5:**
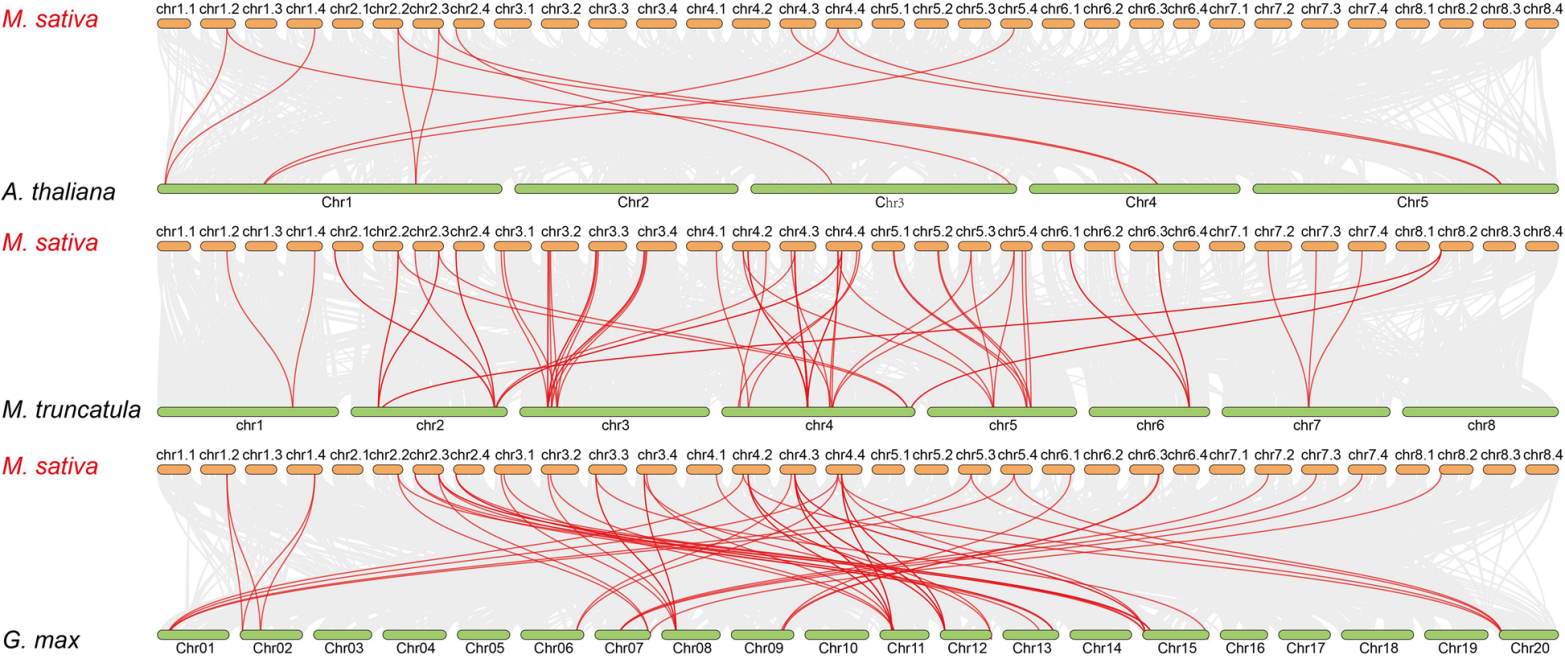
Synteny analysis of *BGLU* genes between *M. sativa* and three representative plant species (*A. thaliana*, *M. truncatula*, and *G. max*). Gray lines in the background represent collinear blocks within *M. sativa* and the indicated plant, while the red lines highlight homologous *BGLU* gene pairs

To further understand the possible evolutionary events of the *BGLU* genes in different groups, three comparative syntenic maps of *M*. *sativa* with *A*. *thaliana*, *M*. *truncatula*, and *G*. *max* were constructed. The results showed 12 collinear *BGLU* gene pairs between *M*. *sativa* and *A*. *thaliana*, 63 collinear *BGLU* gene pairs between *M*. *sativa* and *G*. *max*, and 76 orthologs between *M*. *sativa* and *M*. *truncatula* (Fig. 5, Supplementary Table S4). The number of orthologous gene pairs between *MsBGLUs* and *AtBGLUs* was significantly lower than the number between *MsBGLUs* and *GmBGLUs* or *MsBGLUs* and *MtBGLUs*. This is likely because *M*. *sativa*, *M*. *truncatula*, and *G*. *max* are all legumes.

To explore the evolution type among these duplication events on different chromosomes, the Ka/Ks values were calculated using TBtools software. As shown in Supplementary Table S5, the *MsBGLU10*/*MsBGLU30* gene pairs had a Ka/Ks ratio of 4.96, indicating a high degree of positive selection. However, the Ka/Ks values of 99% of the paralogous *MsBGLU* gene pairs were below 1, implying that the *MsBGLUs* might have undergone a strong purification selection pressure during evolution.

### Analysis of *cis*-acting elements in the promoter regions of *MsBGLU* genes

**Fig. 6:**
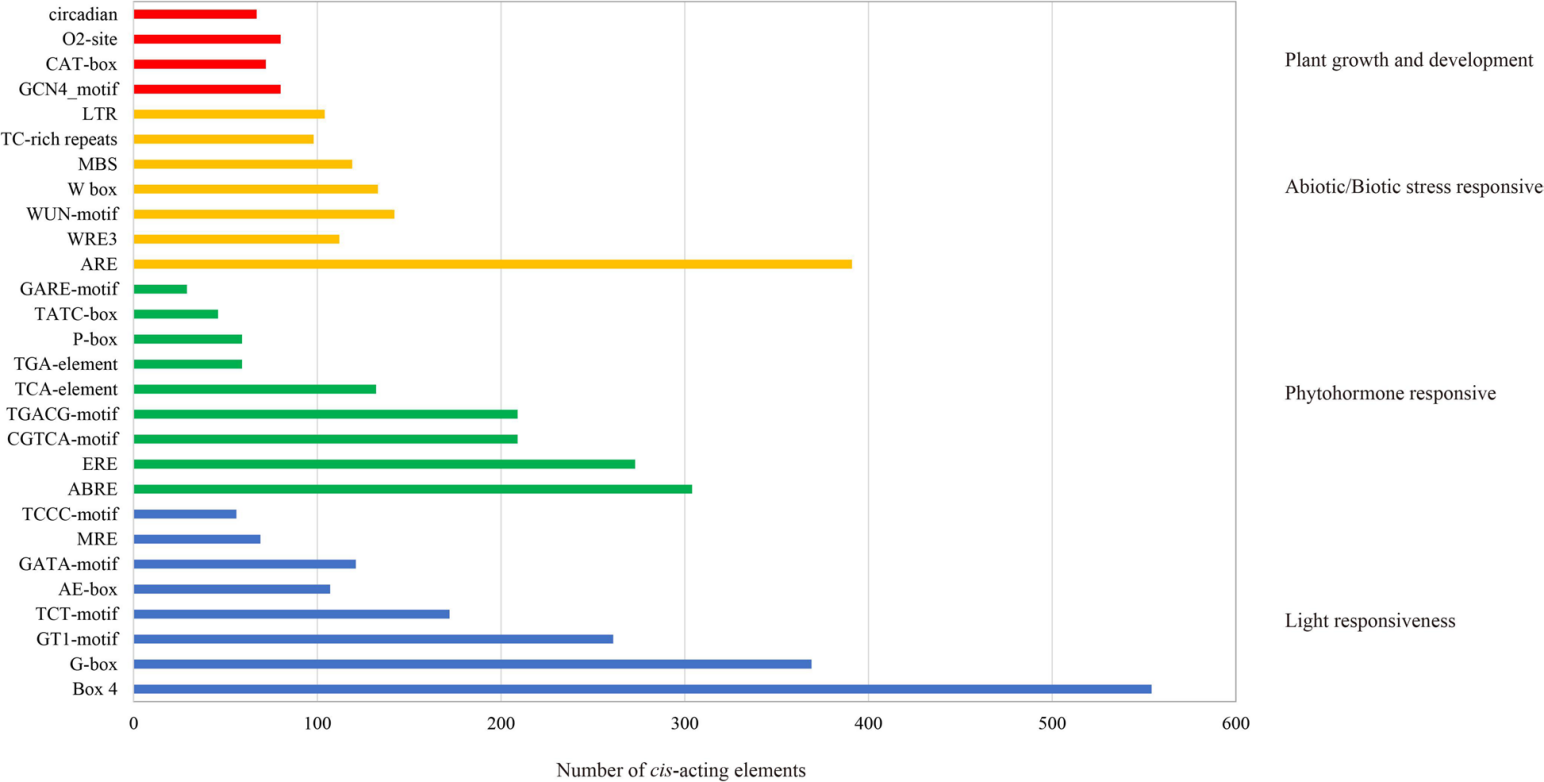
The number of *cis*-acting elements identified in the promoter of *MsBGLUs*. The name of *cis*-acting elements are displayed on the left of the image, and the corresponding function annotation is listed on the right

The *cis*-acting elements in the 2000 bp sequence of the promoter region were analyzed using PlantCARE software. Twenty-eight types of *cis*-acting elements were found associated with responses to stresses and phytohormones in the promoters (Fig. 6, Supplementary Figure S2, Supplementary Table S6). Among plant growth and development clusters, we detected 67 circadian responsive elements (Circadian), 80 O2-site, 72 CAT-box, and 80 GCN4_motif elements. In terms of stress-responsive clusters, we found 104 low-temperature responsive elements (LTR), 98 TC-rich repeats, 119 drought responsive elements (MBS), 133 W-boxes, 254 wound responsive elements (WUN-motif and WRE3), and 391 antioxidant response element (ARE). Among the phytohormone responsive elements, we detected 59 auxin responsive elements (TGA-element), 134 gibberellin responsive elements (P-box, TATC-box, and GARE-motif), 132 salicylic acid responsive elements (TCA-element), 418 MeJA responsive elements (TGACG-motif and CGTCA-motif), 273 ERE (ethylene responses), and 304 ABA responsive elements (Abscisic acid responses, ABRE). Additionally, many light-responsive regulatory elements were identified in the promoter regions of most *MsBGLU* genes (Supplementary Table S6). Taken together, these results suggest that *MsBGLUs* are likely involved in plant growth, development, and abiotic/biotic stress responses.

### Expression analysis of *MsBGLU *genes in alfalfa under abiotic stresses

**Fig. 7:**
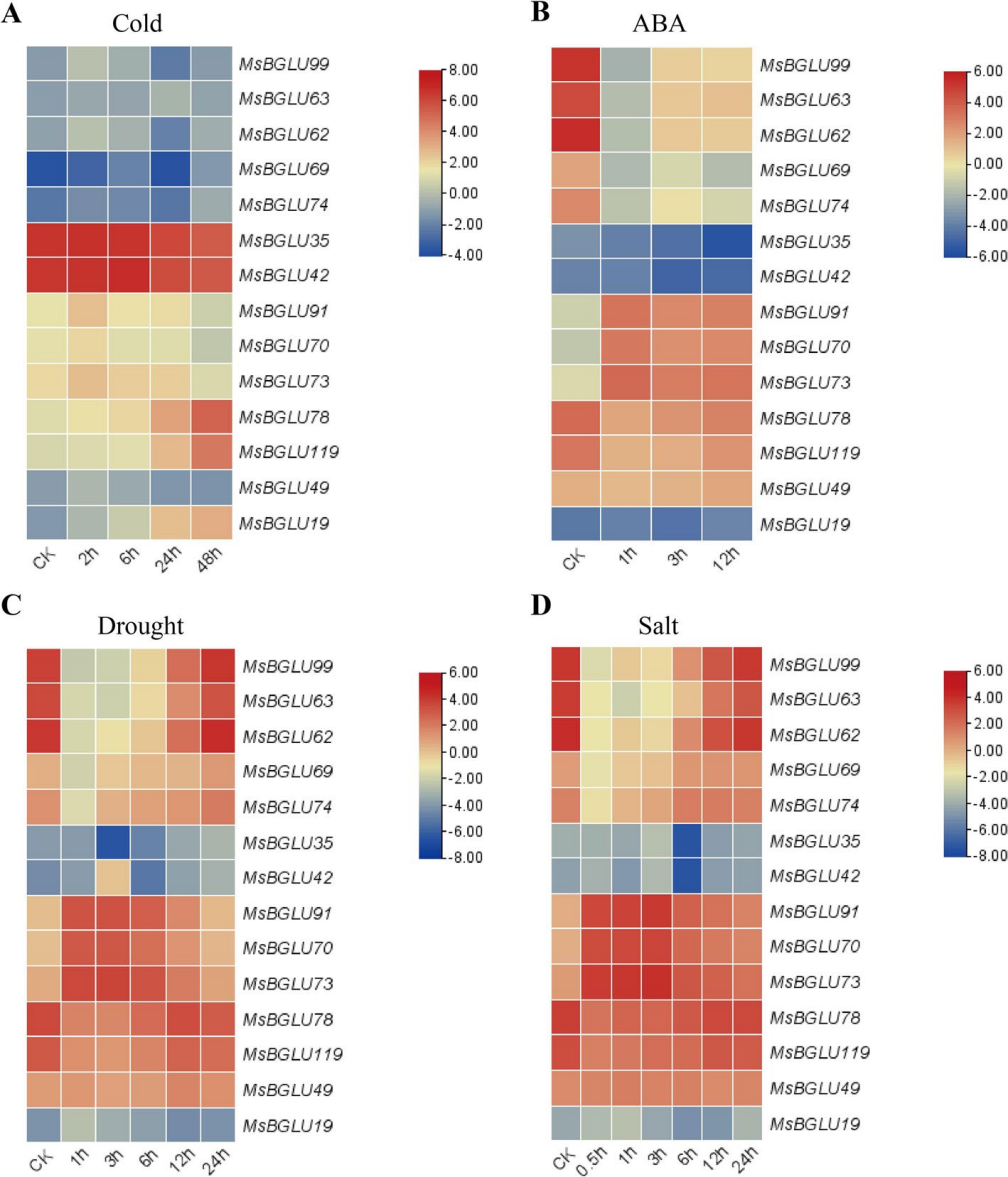
Candidate *MsBGLU* genes responsible for different abiotic stresses. **A** *MsBGLU* genes involved in the cold stress response. **B** *MsBGLU* genes involved in ABA treatment response. **C** *MsBGLU* genes involved in the drought stress response. **D** *MsBGLU* genes involved in the salt stress response

Based on the previous research, we collected transcriptome data under cold, ABA, drought, and salt treatments to clarify the role of the *MsBGLU* genes. The expression levels of these genes were shown in a heatmap (Supplementary Figure S3). The results showed that multiple *MsBGLU* genes were associated with various abiotic stresses. The expression levels of two *MsBGLU* genes (*MsBGLU78* and *MsBGLU119*) were significantly increased under cold stress (Fig. 7A). Under the ABA treatment, the expression levels of five *MsBGLUs* (*MsBGLU99*, *MsBGLU63*, *MsBGLU62*, *MsBGLU78*, *MsBGLU49*) were significantly upregulated, while three (*MsBGLU91*, *MsBGLU70*, *MsBGLU73*) were downregulated (Fig. 7B).

Combining these different treatment results, we also found that multiple *MsBGLU* genes were involved in two or more adversity responses (Fig. 7). *MsBGLU78* and *MsBGLU119* were upregulated in these four treatments. Under ABA, drought, and salt treatment, *MsBGLU99*, *MsBGLU63*, and *MsBGLU62* were upregulated, while *MsBGLU91*, *MsBGLU70*, and *MsBGLU73* were downregulated. Notably, *MsBGLU35* and *MsBGLU42* were only highly expressed under cold stress.

### Validation of the expression profile of *MsBGLU *genes by qPCR

**Fig. 8:**
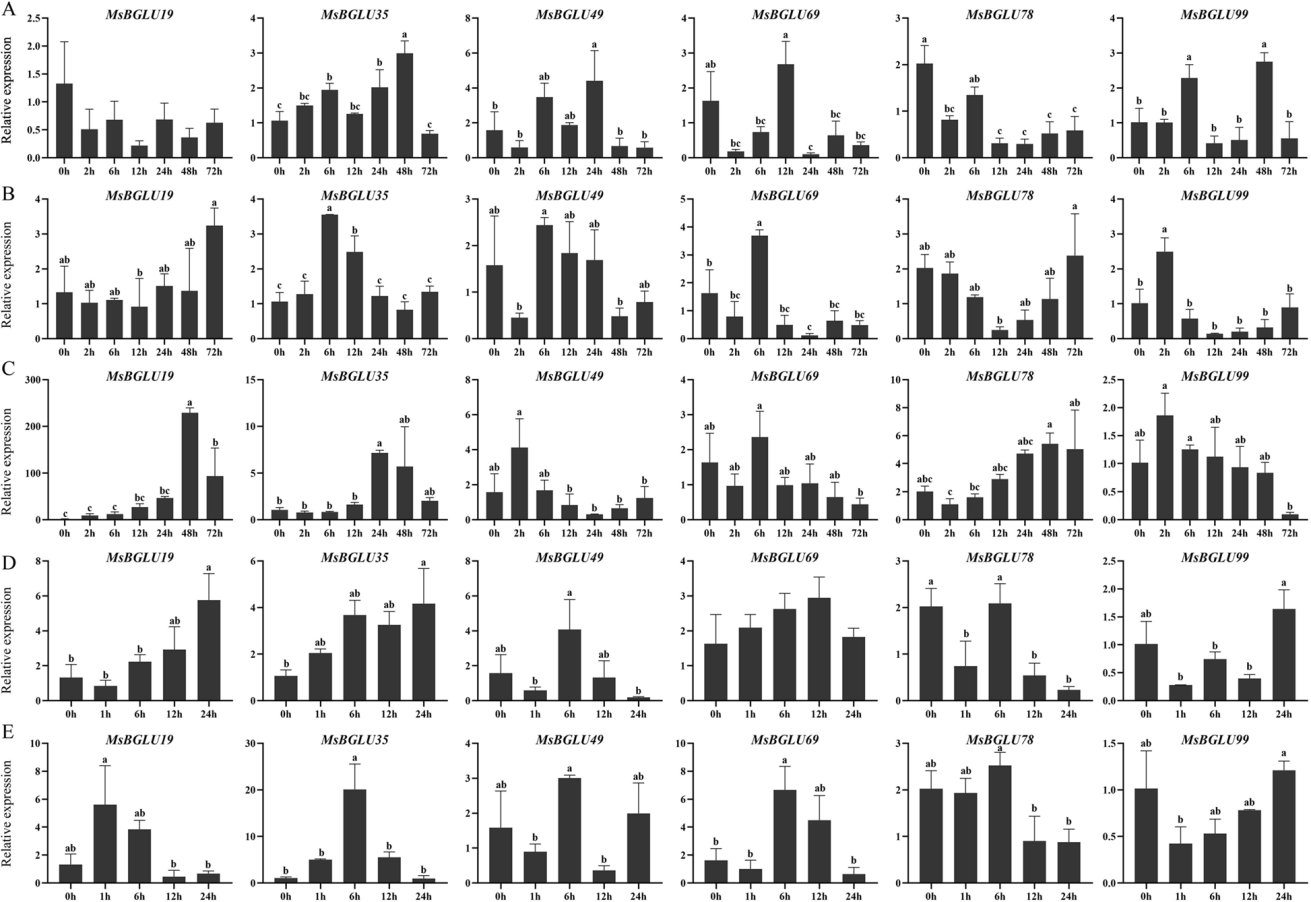
qRT-PCR analysis for 6 representative *MsBGLU* genes in response to (**A**) Mannitol, (**B**) NaCl, (**C**) Cold, (**D**) ABA, and (**E**) IAA treatment. Data were normalized with *MsActin* gene. The vertical bars indicate standard error. Error bars were obtained from three measurements. Lowercase letter(s) above the bars indicate significant differences (α = 0.05, Duncan’s *t*-test among the treatments)

Among all 179 *MsBGLU* genes, six of them (*MsBGLU99*, *MsBGLU69*, *MsBGLU35*, *MsBGLU19*, *MsBGLU78*, and *MsBGLU49*) were highly induced by various stresses. To confirm the result, RT-qPCR was further performed. As shown in Fig. 8, the expression profiles of the six candidate genes were basically consistent with RNA-Seq results. Under cold stress (Fig. 8C), the expression levels of *MsBGLU19* and *MsBGLU78* were significantly up-regulated at 48 h and then decreased; the expression levels of *MsBGLU49* and *MsBGLU99* were significantly up-regulated at 2 h and then decreased gradually. The expression level of *MsBGLU69* reached the highest at 6 h, and *MsBGLU35* was comparatively higher at 24 and 48 h. These results were consistent with the above RNA-seq data (Fig. 7). Under mannitol stress (Fig. 8A), *MsBGLU35* was significantly up-regulated to a certain level. *MsBGLU78* was obviously down-regulated during the late treatment stages. *MsBGLU49*, *69*, and *99* reached the highest at 24 h, 12 h, and 48 h, respectively. However, the expression level of *MsBGLU19* was not changed significantly. Under NaCl stress (Fig. 8B), the expression level of *MsBGLU19* increased to 72 h gradually, whereas *MsBGLU35*, *69* were significantly up-regulated at 6 h and then decreased gradually. The expression level of *MsBGLU99* reached the highest at 2 h. Besides, NaCl had no significant effect on the expression levels of *MsBGLU49* and *MsBGLU78*. Under ABA treatment (Fig. 8D), the expression levels of *MsBGLU19* and *MsBGLU35* were greatly elevated. Additionally, *MsBGLU78* was remarkably diminished after 12 h of ABA treatment, whereas ABA showed no evident effect on the expression levels of *MsBGLU49*, *69*, and *99* genes. For IAA treatment (Fig. 8E), the expression levels of *MsBGLU35* and *MsBGLU69* were only up-regulated after 6 h. *MsBGLU19*, *49*, *78*, and *99* showed no significant increment in response to the IAA supplement. Collectively, the results confirm that these *MsBGLU* genes was most likely key *BGLU* genes involved in response to stress and hormone stimuli in *M. sativa*.

## Discussion

β-glucosidase genes play diverse and important roles in response to abiotic stress and plant development [27], including roles in cell-wall oligosaccharides, lignification, activation of phytohormones, defense, secondary metabolism [12, 16]. It is noteworthy that *BGLUs* participate in most of these above processes through an effective mechanism, namely hydrolyzing (activating) inactive and stable glucoside compounds, and increasing cellular ABA levels to protect plants from various stresses. Because of their important roles, *BGLU* genes have been identified and functionally analyzed genome-wide in many plant species. However, a systematic analysis and in-depth study of the *BGLU* genes at the whole-genome level is still lacking in alfalfa, because of its autotetraploid and difficulty in genome assembly [25]. In this study, a total of 179 *MsBGLU* gene members were identified in the alfalfa reference genome (Supplementary Table S1). The number of *BGLU* genes in alfalfa was higher than those in *A. thaliana* (*n* = 47) [28], *O. sativa* (*n* = 40) [29], *Zea mays* (*n* = 26) [30], *M. truncatula* (*n* = 51) [26], and *Brassica rapa* (*n* = 64) [14]. That is mainly due to the fact the genome of ‘XinJiangDaYe’ is an allele-aware chromosome-level genome consisting of 32 allelic chromosomes. Hence, *MsBGLU* genes identified include a portion of allelic genes. Another reason for the increase in *MsBGLU* numbers could be gene duplication events.

It is well known that gene duplication plays a vital role in the rapid expansion and evolution of gene families [31]. In this study, 143 pairs of segmental duplication genes and 13 pairs of tandem duplication were detected in the alfalfa *BGLU* gene family, supporting the inference that *MsBGLU* gene family expands through segmental and tandem duplication events, and the former played a predominant role. This observation is in accordance with *PtBGLU* genes [27]; conversely, the evolution of *MtBGLU* genes was mainly driven by tandem duplication [26]. It is not difficult to speculate that the number of gene duplications is related to the number of chromosomes and family/subfamily members. Synteny maps among the three representative species (*A*. *thaliana*, *M*. *truncatula*, and *G*. *max*) and alfalfa were constructed to better understand the phylogenetic relationships. There were only 12 gene pairs between *MsBGLU* and *AtBGLU*, but 63, and 76 gene pairs between *MsBGLU* and *GmBGLU*, and *MtBGLU*, respectively (Fig. 5, Supplementary Table S4). It is likely that *M. sativa*, *G. max*, and *M. truncatula* belong to the leguminous family, while *A. thaliana* belongs to the cruciferous family. There were relatively more collinear gene pairs between *M. sativa*, *G. max*, and *M. truncatula*, indicating a closer genetic relationship. Especially, the genetic relationship between *M. sativa* and *M. truncatula* is closer than that of *G. max*, because both of them belong to the same genus of *Medicago*.

On the basis of functional characterization of homologs in different plant species, gene functions were analyzed and predicted by phylogenetic tree analysis. *MsBGLU19*/*25*/*26* were clustered in cluster IV with *AtBGLU45*/*46* (Fig. 1), which hydrolyzed lignin precursors in *A. thaliana* [17]. This suggests that *MsBGLU19*/*25*/*26* may be involved in lignin metabolism in *M. sativa*. As glucosyltransferase, *AtBGLU6* and *AtBGLU10* in cluster V play an important role in the biosynthesis of flavonoid glycosides in *A. thaliana* [28, 32]. It is speculated that the *BGLUs* of cluster V in *M. sativa* may have the similar function of synthesise flavonoid glycosides to resist abotic stress. Notably, cluster I contained more than half (108/179) of *MsBGLU* family members and twelve members of *A. thaliana*. The genes of this cluster were mainly expressed in roots that accumulate natural isoflavonoids [33], which participate in plant-microbe interactions, such as symbiotic or defensive mechanisms against pathogens infection. In *M. truncatula*, four *BGLUs* participate in methyl jasmonate (MJ) signal-induced hydrolysis of stored isoflavone glucosides for phytoalexin medicarpin accumulation [26]. Therefore, it is inferred that the *BGLUs* of cluster I in *M. sativa* may have the similar function of hydrolyzing isoflavonoid glucosides to defensive aglycones under adverse stresses. Besides, 16 out of 47 *AtBGLUs* were grouped into the cluster At_I. Actually, orthologous genes of this cluster are also present in other species of *Brassicales* order, but not in closely related *M. sativa* and *M*. *truncatula* counterparts (At I) in Fig. 1, indicating they might have diverged before *M. sativa* and *A. truncatula*. Similarly, eight *MsBGLUs* in cluster II have no closely related *A. thaliana* homologs. Interestingly, in each group (except for At_1), *MsBGLUs* have homologous genes of *MtBGLUs*, likely due to the relatively close evolutionary distance between *M. sativa* and *M*. *truncatula*.

Many studies provide evidence that *BGLU* genes are involved in response to various plant abiotic stresses [17, 29, 34]. For instance, *AtBGLU18* with an ER retention signal is induced by dehydration and contributes significantly to drought resistance in *A*. *thaliana* [20]; chloroplast-localized *Os3BGLU6* is responsive to drought and ABA treatments, and disruption of *Os3BGLU6* in rice results in lower ABA content and higher stomatal density [35]; *MtBGLU21*, *MtBGLU22*, *MtBGLU28*, and *MtBGLU30* are strongly induced by abiotic stresses and hormone treatments, indicating their important roles in development and stress tolerance [26]. Alfalfa has high tolerance to abiotic stress, but the role of *MsBGLUs* in abiotic stress has not been clarified. In the present study, we found that *MsBGLU78* was homologous gene of *MtBGLU19*, while *MsBGLU69* and *MsBGLU99* were homologous genes of *MtBGLU28* and *MsBGLU21*, respectively (Fig. 1). The expression levels of *MsBGLU19* and *MsBGLU35* were found to be induced by cold stress (Fig. 8). Therefore, based on phylogenetic analysis and expression analysis, we inferred that *MsBGLU19*, *35*, *69*, *78* and *99* may perform the function of regulating resistance to abiotic stress in alfalfa. Furthermore, there were plentiful stress and hormone responsive elements detected in the promoter regions of *MsBGLUs*, and the expression patterns of most *MsBGLU* genes exhibited differential responses to various abiotic stresses at the transcriptional level, indicating the functional diversity of them. The qRT-PCR quantitative data showed that the expression of *MsBGLU69* was induced by IAA, and *MsBGLU99* was induced by mannitol and NaCl to some extent; the previous studies showed that their homolog *MtBGLU28* and *MtBGLU21* in *M. truncatula* respectively were induced by IAA, ABA, SA and GA3 treatments [26], indicating their potential roles in various signaling network. Additionally, the expression of *MsBGLU35* was induced not only by abiotic stresses but also by hormone treatments, and *MsBGLU19* was induced by cold stress and ABA treatment, indicating the important roles against stresses (Fig. 8). Besides, root-specific *AtBGLU42* has been known to enhance the ability of beneficial rhizosphere bacteria to stimulate the immune system, while regulating the iron deficiency response in *A*. *thaliana* roots [36], and its homolog *MsBGLU49* in alfalfa was induced by mannitol stress (Fig. 8), indicating the diversified functions between *AtBGLUs* and *MsBGLUs*. Previous study also showed that *MtBGLU19* was induced by NaCl stress [26], but the expression level of its homolog *MsBGLU78* was decreased by NaCl stress to 12 h, then increased gradually. The different expression patterns exhibited among *MsBGLUs* under stress may be due to their different defense mechanisms, and further in-depth research is needed.

Alfalfa, as one of the most important forage crops in the world [37], often suffers from various adverse effects like drought and salt stress, resulting in yield and quality loss [3]. The *BGLU* gene family is involved in plant growth, development, and abiotic stress response. Therefore, it is important to mine *MsBGLU* genes related to stress tolerance and develop molecular breeding strategies for the genetic improvement of alfalfa. Our genome-wide identification and expression analysis of *MsBGLU* genes provide knowledge of candidate genes of abiotic stress resistance in alfalfa, which may be helpful for better understanding the biological roles of *MsBGLU* genes and for the further functional analysis of them.

## Conclusion

In this study, a comprehensive and systematical investigation of *GH1* β-glucosidases in *M*. *sativa* was carried out. A total of 179 *MsBGLUs* were identified from autotetraploid cultivated alfalfa genome. They were classified into five main clusters, with highly identical amino acid sequences and motif compositions. The conserved domain, gene structure, genomic location, *cis*-element, and expression pattern were conducted. The *cis*-acting element responsive to plant hormones (ABA, auxin, MeJA, salicylic acid, and gibberellin) and stresses were found in the promoter of some *MsBGLUs*. Moreover, expression levels of *MsBGLUs* were analyzed in response to various treatments (drought, salt, ABA, and cold) based on available RNA-seq data and qPCR validation, indicating that *MsBGLUs* played important roles in *M*. *sativa* stress tolerance, especially *MsBGLU19*, *MsBGLU35*, *MsBGLU49*, *MsBGLU69*, *MsBGLU78*, and *MsBGLU99*. Overall, the study provides valuable resources for understanding the potential molecular function of *MsBGLU* genes, and lays a foundation for further improvement of stress resistance and hormone signaling transduction in alfalfa.

## Materials and methods

### Plant materials, growth conditions, and stress treatments

The alfalfa cultivar ‘XinJiangDaYe’ used in this experiment was obtained from College of Grassland and Environment Sciences of Xinjiang Agricultural University. First, the seeds that were full and uniform in shape were selected and sterilized in 1% NaClO solution for 10 min. Then they were placed onto two layers of filter paper moistened with distilled water in circular Petri dishes in the dark at 22 °C for 3 days. Then, seedlings were transplanted to square plastic pots with vermiculite in a greenhouse for growth (16/8 h light/dark photoperiod, 25 °C/23°C day/night temperature, 52% relative humidity). The 6-week-old alfalfa seedlings were subjected to different treatments. For cold stress, alfalfa seedlings were transferred to an artificial climate chamber at 10 °C. For drought stress, plants were irrigated with 400 mM mannitol solution to simulate drought stress. For salt stress, 300 mM NaCl was used to simulate salt stress. Seedling leaves were collected at seven time points (0, 2, 6, 12, 24, 48, and 72 h), including three biological replicates, and the samples treated for 0 h were used as controls. For phytohormone treatments, plants were treated with 100 µM abscisic acids (ABA) and 100 µM auxin (IAA), respectively. Seedling leaves were collected at six time points (0, 1, 6, 12, 24, and 48 h). All samples were immediately frozen in liquid nitrogen and stored at − 80 °C before RNA extraction.

### Identification of *MsBGLU *genes of *GH1* family in alfalfa reference genome

The reference genome sequence (Xinjiangdaye) of alfalfa was downloaded from Figshare (https://figshare.com, accessed on 12 November 2022). To more comprehensively investigate the *BGLU* genes, the BGLU protein sequences of *A*. *thaliana* were obtained from genome databases of *A*. *thaliana* (TAIR, http://www.arabidopsis.org/, accessed on 1 March 2023); the BGLU protein sequences of *M*. *truncatula*, a model plant for legumes, were acquired as described by Yang et al. [26]; the reference genome of *G. max* (Wm82.a4.v1) was downloaded from the Pytozome (https://phytozome-next.jgi.doe.gov/). The *BGLU* candidate members of *GH1* family (*BGLU* was referred as *GH1* family hereinafter) of alfalfa were carried out by two steps. First, a hidden Markov model (HMM) profile of Glyco_hydro_1 (PF00232) was downloaded from the Pfam database (http://pfam.xfam.org/) and used as the query (*P* < 1e^−10^) to search the *M*. *sativa* protein sequence data with HMMER3 software using default parameters [38]. Second, BGLU protein sequences of *A*. *thaliana* and *M*. *truncatula* were utilized as the query sequences for BLAST analysis against the cultivar XinJiangDaYe genome database with an E-value cutoff 1e^−10^. After removing all of the redundant sequences, the candidate MsBGLU protein sequences were screened via NCBI Batch CD-search (https://www.ncbi.nlm.nih.gov) to confirm the conserved BGLU domain. Finally, the physicochemical properties of *MsBGLUs* including molecular weight (MW) and theoretical isoelectric points (pI) were analyzed using ProtParam (http://web.expasy.org/protparam/, accessed on 6 May 2023). The subcellular localization of *MsBGLUs* was predicted using the WoLF PSORT (https://wolfpsort.hgc.jp/, accessed on 6 May 2023).

### Multiple sequence alignment, phylogenetic analysis, and classification of the *MsBGLU *genes

In order to analyze the evolutionary relationships of the obtained *BGLUs* in alfalfa, the BGLU protein sequences of *M. sativa*, *A. thaliana*, and *M. truncatula* were used for multiple sequence alignment with Muscle [39] and trimmed automatically with TrimAL [40]. Subsequently, an unrooted phylogenetic tree was constructed based on the neighbor-joining (NJ) method with 1000 replicated bootstrap values using MEGA 7.0 software. According to the classification of *MtBGLU*, all the identified *MsBGLU* genes were divided into 5 groups [26]. The online program iTOL (https://itol.embl.de/, accessed on 7 May 2023) was used to optimize the evolutionary tree [41].

### Gene structure and motif analysis of *MsBGLU *genes

Understanding the gene structure can help reveal gene functions, regulation, and evolution. To visualize the exon-intron structure of *MsBGLU* genes, the gene structure was displayed using Tbtools [42], according to the alfalfa genome annotation file [25]. Conserved motifs of MsBGLU proteins were identified using the MEME program (http://meme-suite.org/tools/meme) with parameters of maximum of 10 motifs and a range of motif width 6 to 200 and displayed using TBtools software [42].

### Chromosomal locations, gene duplication, and syntenic analysis with several plant species

To better recognize the genomic distribution of *MsBGLU* genes, a chromosomal location map of the *MsBGLU* genes was drafted based on the alfalfa genome annotation files using TBtools [42]. A multiple Collinearity Scan toolkit (MCScanX) was adopted to analyze the duplication events of alfalfa *BGLU* genes with default parameters [43].

Gene duplication is a major source to produce new genes, which leads to the proliferation and diversification of TFs genes in the plant kingdom. To exhibit the interspecific synteny relationship between alfalfa and the other three representative model plant species (*A. thaliana*, *M. truncatula*, and *G*. max), the syntenic maps were constructed using Dual Synteny Plotter of TBtools software [42].

### *Cis*-regulatory element analysis in the promoter region of *MsBGLU* genes

A thorough analysis of the *cis*-regulatory elements in *MsBGLU* genes may provide further insight into their function. The *cis*-regulatory elements in the upstream 2000 bp promoter sequences were submitted to the online program PlantCARE database (http://bioinformatics.psb.ugent.be/webtools/plantcare/html/, accessed on 6 May 2023) [44].

### Expression analysis of *MsBGLU* genes under abiotic stresses

In previous studies, to acquire additional genetic information of alfalfa under abiotic stresses, RNA-Seq projects of alfalfa under cold (SRR7091780-SRR7091794), drought, salt, and ABA treatments (SRR7160313-SRR7160357) were performed [22]. In this study, to realize the roles of *MsBGLU* genes in response to abiotic stresses, the clean reads were mapped to the “Xinjiangdaye” genome by using Hisat2 software [31]. The gene expression level was estimated by using FPKM value (fragments per kilobase of transcript per million reads mapped). Subsequently, TBtools software was used for data visualization [43].

### Expression analysis by qRT-PCR

Six *MsBGLU* genes were selected for qRT-PCR experiments to validate the RNA-Seq data. Total RNAs were extracted using Eastep® Super total RNA Extraction kit (Promega, Shanghai, China) according to the manufacturer’s instructions. Subsequently, a NanoDrop ND1000 spectrophotometer (Thermo Scientific, Waltham, MA, USA) was applied to determine the concentration of each sample. The first-strand cDNA synthesis was performed using HiScript®III RT SuperMix for qPCR (+ gDNA wiper) (Vazyme Biotech, Beijing, China) according to the manufacturer’s instructions. The expression patterns of *MsBGLU* genes were explored by using the SYBR Green RT-PCR Kit (Takara). The reaction was conducted as follows: 94 °C for 30 s, followed by 45 cycles of 94 °C, for 5 s, and 60 °C for 34 s. Each reaction was carried out in three biological repetitions, and the relative expression was calculated using the 2^−∆∆Ct^ method. The results were analyzed by means ± SE. The *Medicago actin* gene was amplified as the reference gene to normalize the amount of the template. The primer sequences used in this study were shown in detail in Supplementary Table S1. The specific primers for qRT-PCR were designed using the Primer Premier 5 software and listed in Supplementary Table S7.

### Supplementary Information

**Additional file 1: Table S1.** Protein property of MsBGLU proteins. **Table S2.** Information for each MsBGLU motif. **Table S3.** Number of homologous gene pairs. **Table S4.** One-to-one orthologous relationships between *M.sativa*, *A. thaliana*, *G.max* and *M.truncatula*. **Table S5.** The BGLU protein sequence in *Medicago sativa*. **Table S6.** Name and position of *cis*-acting elements in *MsBGLUs*. **Table S7.** List of primers used in this research. **Fig. S1.** Phylogenetic relationships, conserved patterns, and gene structure of *BGLU* in *M. sativa*. The motifs, numbered 1-10, are displayed in different colored boxes. **Fig. S2.** Analysis of *cis*-acting elements in the putative promoter of *MsBGLUs*.

## Data Availability

RNA-seq raw data used in this study was downloaded from the SRA database in NCBI. It could be accessed with accession numbers PRJNA450305 and PRJNA454564. PlantCARE database (http://bioinformatics.psb.ugent.be/webtools/plantcare/html/, accessed on 6 May 2023).
