# Supplementary material for: Genome-wide identification and expression analysis of the glycosyl hydrolase family 1 genes in Medicago sativa revealed their potential roles in response to multiple abiotic stresses
Source: BMC Genomics. 2024 Jan 2;25:20. doi: 10.1186/s12864-023-09918-w (PMC10759430; doi:10.1186/s12864-023-09918-w)
Supplement: Supplementary file 1 — Additional file 1: Table S1. Protein property of MsBGLU proteins. Table S2. Information for each MsBGLU motif. Table S3. Number of homologous gene pairs. Table S4. One-to-one orthologous relationships between M.sativa, A. thaliana, G.max and M.truncatula. Table S5. The BGLU protein sequence in Medicago sativa. Table S6. Name and position of cis-acting elements in MsBGLUs. Table S7. List of primers used in this research. Fig. S1. Phylogenetic relationships, conserved patterns, and gene structure of BGLU in M. sativa. The motifs, numbered 1-10, are displayed in different colored boxes. Fig. S2. Analysis of cis-acting elements in the putative promoter of MsBGLUs. [file 12864_2023_9918_MOESM1_ESM.zip › Additional File 1/SupFigure.docx]

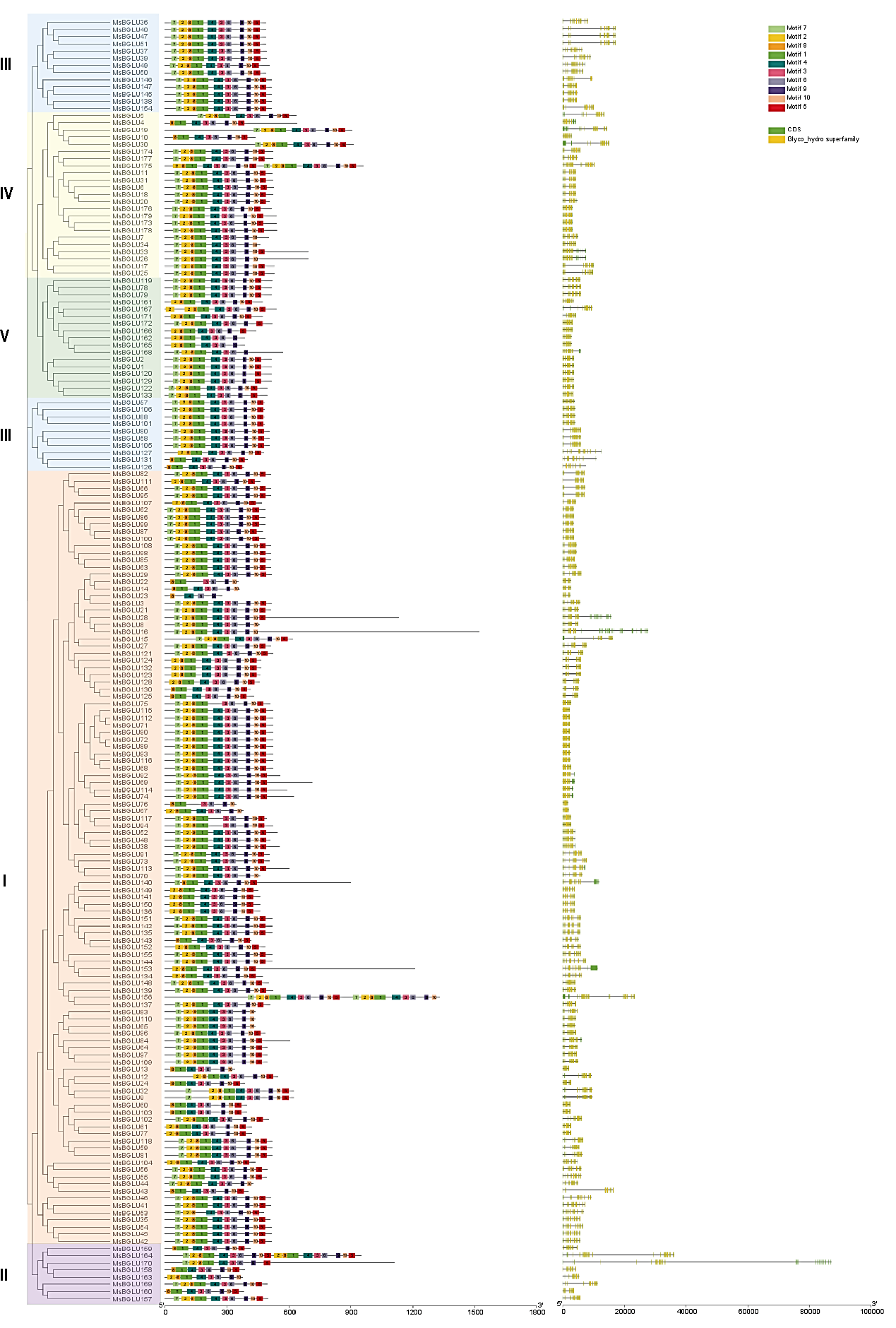


Fig. S1 Phylogenetic relationships, conserved patterns, and gene structure of *BGLU* in *M. sativa*. The motifs, numbered 1-10, are displayed in different colored boxes.


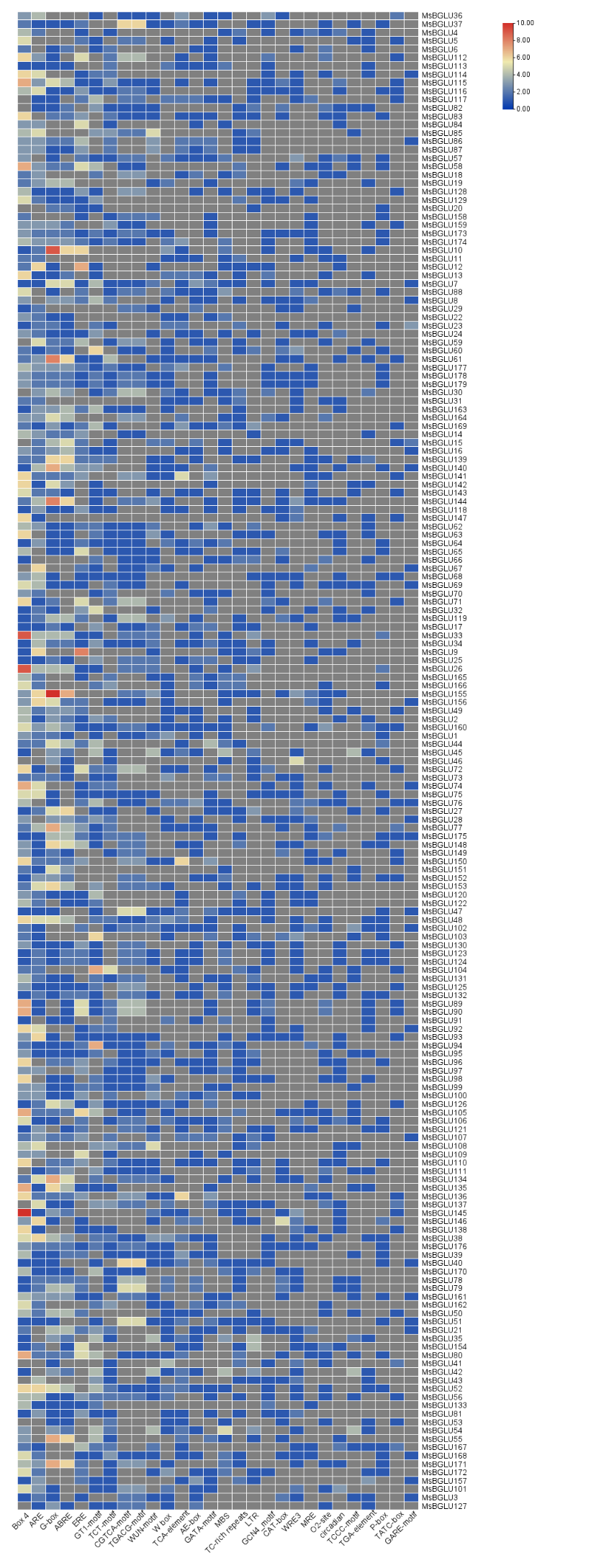


Fig. S2 Analysis of *cis*-acting elements in the putative promoter of *MsBGLUs*.


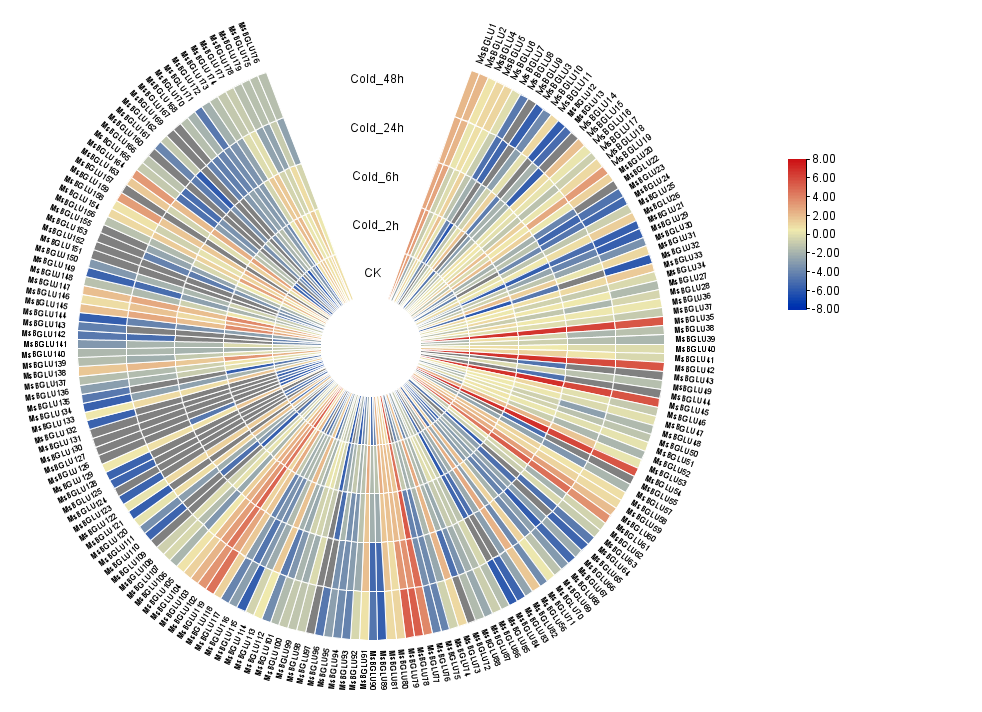


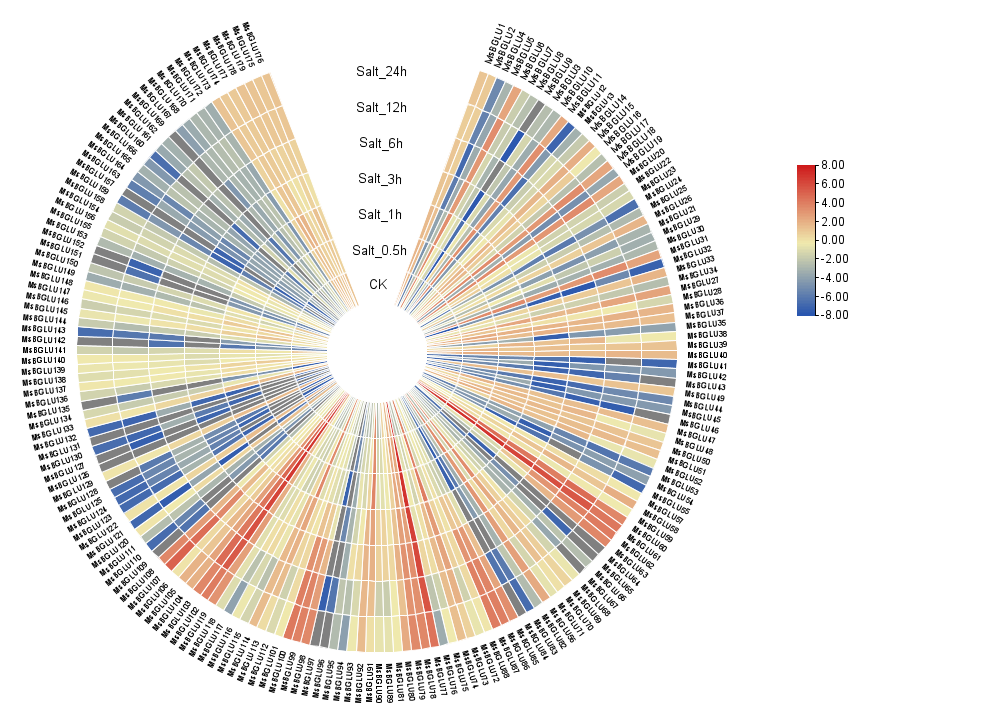


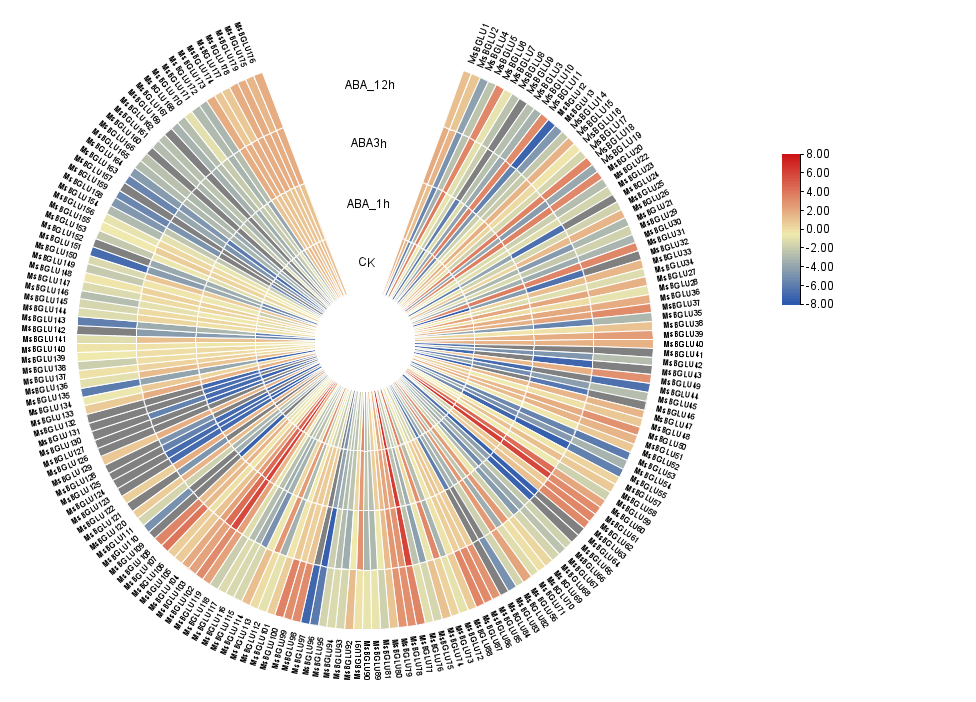


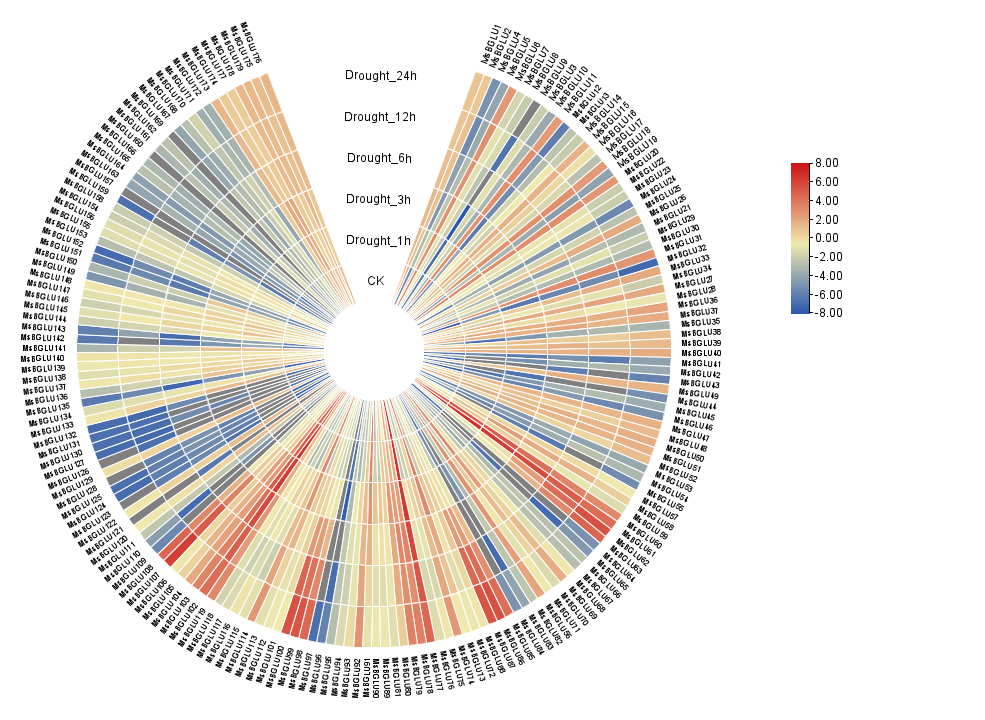


Fig. S3 Heatmap of *MsBGLUs* expression in response to abiotic treatment (cold, salt, drought stress and ABA treatment). The FPKMs were calculated for expression values from RNA-Seq data. The relative expression levels are log2-transformed and visualized for heatmap. The color of circles from blue to red shows the expression level form negative to positive values after normalization.
